# Thoracoscopic segmentectomy for intralobar sequestration in adult: a case report

**DOI:** 10.1186/s40792-018-0427-2

**Published:** 2018-06-07

**Authors:** Naoko Ose, Yukiyasu Takeuchi, Yuko Kobori, Akio Hayashi, Daieuke Ishida, Teruka Kawai

**Affiliations:** 1 Department of General Thoracic Surgery, Osaka University Graduate School of Medicine, 2-2 Yamadaoka Suita-shi, Osaka, Japan; 2 Department of General Thoracic Surgery, National Hospital Organization Toneyama Hospital, 5-1-1 Toneyama Toyonaka-shi, Osaka, Japan

**Keywords:** Segmentectomy, Sequestration, Thoracoscopic surgery, Adult

## Abstract

**Background:**

Surgical resection is the first choice for intralobar sequestration (ILS). A lobectomy is often performed, though we consider that a segmentectomy may be sufficient for benign cases if the sequestration is completely included within a segment.

**Case presentation:**

We treated a 36-year-old female diagnosed with ILS. Chest computed tomography (CT) revealed several cystic lesions with niveau formation and consolidation in left segment (S)10 without communication of the bronchus and abnormal artery branching from the descending aorta. We performed a sublobar resection of left S10 including sequestration through a thoracoscopic minimally sized incision. The sequestration had dark red appearance and was completely included in the S10. The boundary line of S10 was clear with inflation of the lung after cutting bronchus 10. The postoperative course was uneventful. Chest CT findings at 2 years after surgery showed good expansion of the residual left lower lobe with no consolidation and respiratory function were nearly the same as the preoperative condition.

**Conclusions:**

A thoracoscopic segmentectomy for ILS is a feasible and useful procedure for qualified cases, even in adult patients who had repeated inflammation.

## Background

Surgical resection is the first choice for intralobar sequestration (ILS). On the other hand, a lobectomy is often performed because of several reasons. For example, it is considered that the adjacent normal lung have damage due to repeated inflammation associated with sequestration, and/or ILS, which most likely occur in young patients who have adequate respiratory function. However, in cases of benign disease, we consider that a segmentectomy may be sufficient if sequestration is completely included in a segment by minimally invasive incision. There are some reports of a segmentectomy for from an infant to adult, but a few especially with thoracoscopic approach. This is the report of a thoracoscopic segmentectomy of a left segment (S)10 in an adult patient.

## Case presentation

**Fig. 1:**
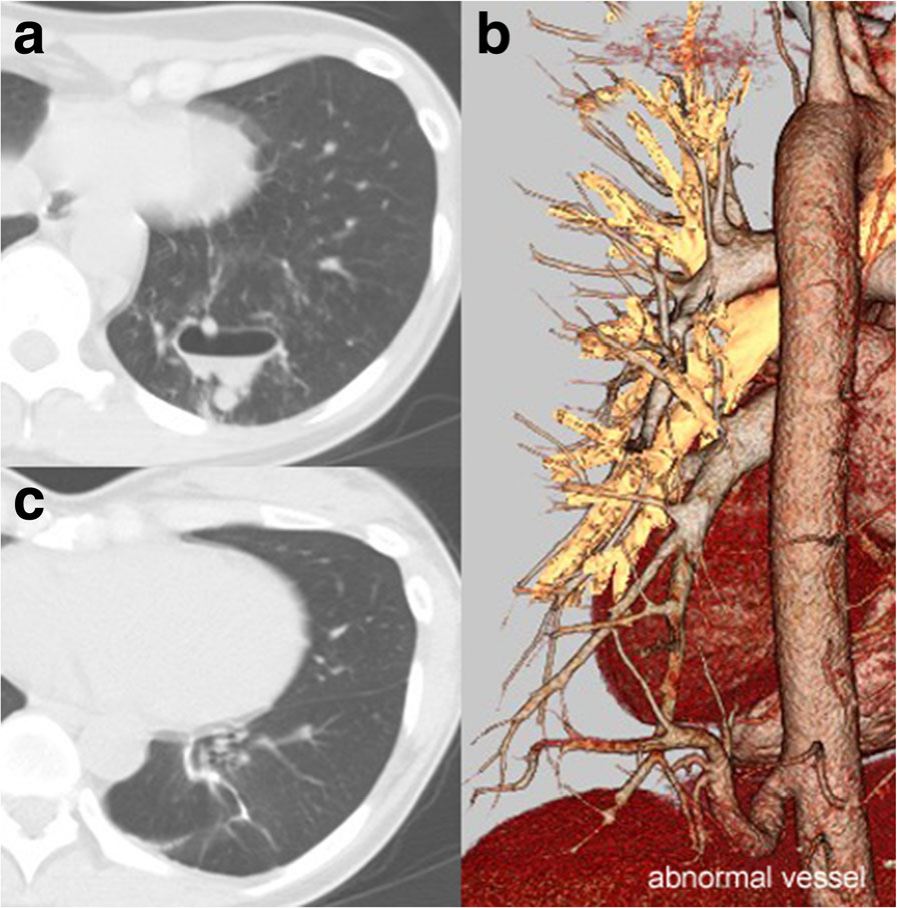
CT images obtained before and after treatment. **a** Chest CT scan showing several cystic lesions with niveau formation and consolidation in left S10 without communication with the bronchus. **b** Three-dimensional CT scan showing an abnormal artery branching from the descending aorta and normal pulmonary vein. **c** Chest CT scan obtained at 2 years after surgery showing good expansion of residual left lower lobe with no consolidation

The patient was a 36-year-old female with no symptoms to reveal consolidation in the left lower lung shown by chest radiography performed as part of a medical examination, though the patient did report long-lasting cold symptoms like cough and sputum. Chest computed tomography (CT) showed several cystic lesions with niveau formation and consolidation in left S10 (Fig. 1a), without communication with the bronchus. Three-dimensional CT revealed an abnormal artery branching from the descending aorta and a normal pulmonary vein (Fig. 1b); thus, our diagnosis was ILS. Laboratory findings were normal without evidence of inflammation.

**Fig. 2:**
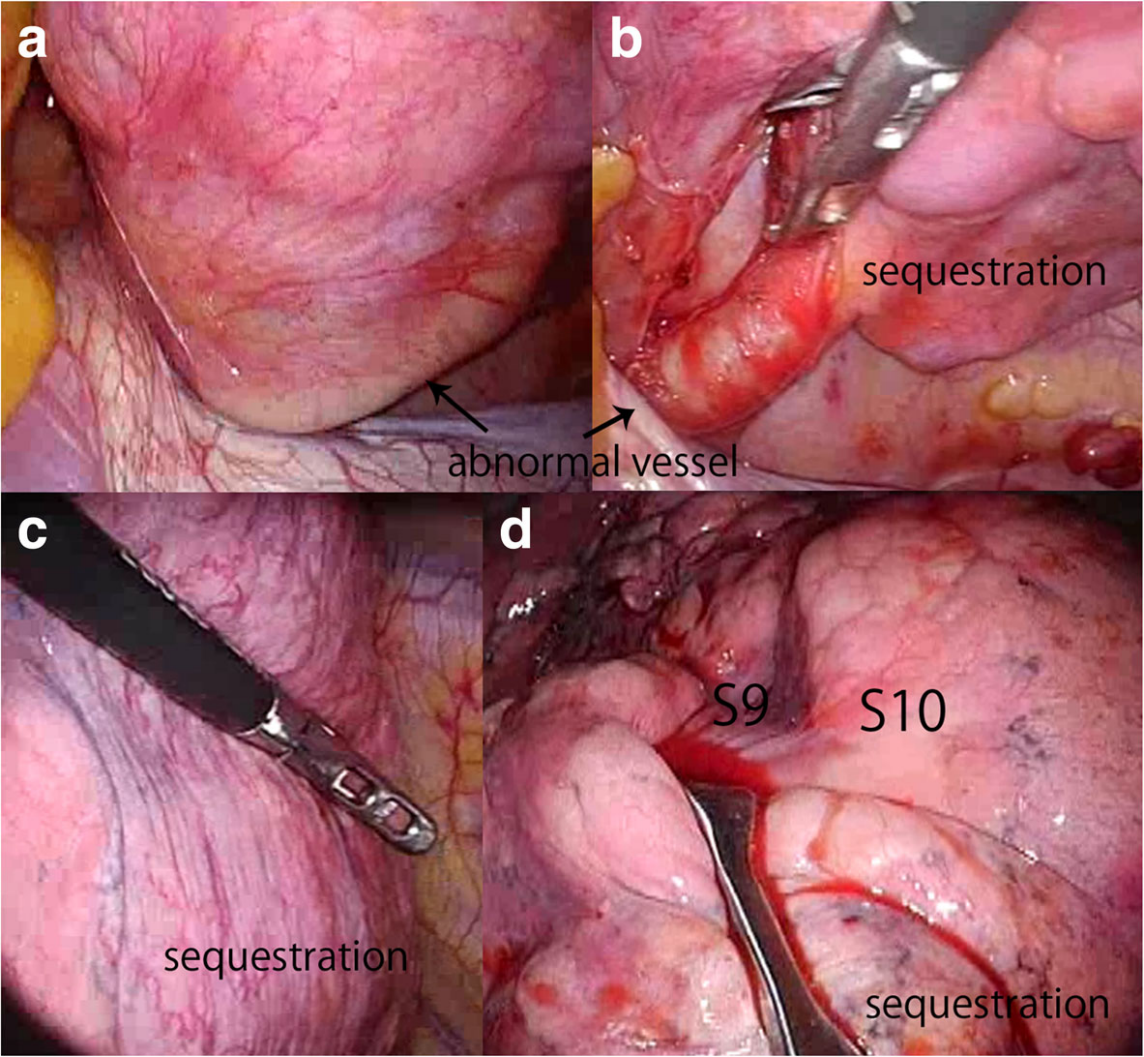
Intraoperative findings. **a** The abnormal artery was inside the lung ligament **b** branching from the descending aorta. **c** The sequestration had a dark red appearance due to developed capillary vessel of visceral pleura. **d** The sequestration portion was completely included in S10. The boundary line was clearly revealed with inflation of the lung after cutting B10

Because of patient desire to preserve pulmonary function for future childbearing, we conducted a sublobar resection of left S10 including sequestration through a thoracoscopic minimally incision (one window, three ports). The window, which length was 4 cm, was placed on middle axillary line in the fifth intercostal spaces, and three ports, two were 5 mm and one was 12 mm in size, were on the anterior axillary line in the sixth intercostal spaces and the middle and posterior axillary line in the seventh intercostal spaces. An abnormal artery was found inside of the lung ligament branching from the descending aorta (Fig. 2a, b). It was cut after double pierced ligature with silk blade. The boundary line was detected between S6 and basal segment by selective inflation from bronchus (B)6 and divided the parenchyma with stapler. The sequestration had a dark red appearance due to developed capillary vessel of visceral pleura (Fig. 2c) and was completely included in S10 (Fig. 2d). B10 was identified easily from the intersegmental space after division of lower lobe into S6 and basal segments.

The boundary line was clear when inflation of the lung was performed after cutting B10. The parenchyma was divided with a stapler.

**Fig. 3:**
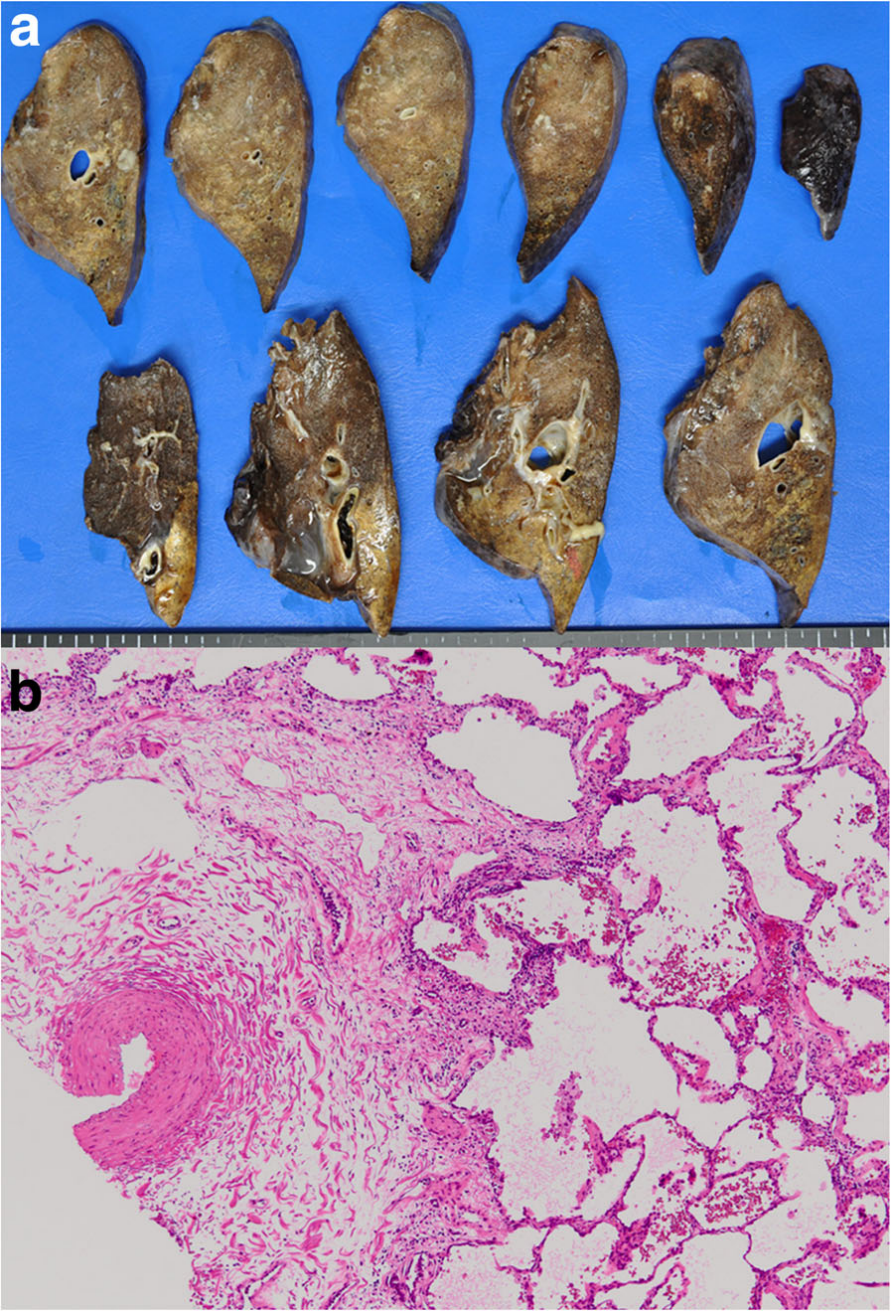
Histopathological findings. **a** Macroscopic specimen showing multiple cystic lesions with a boundary and elastic artery flow into that area. **b** Hematoxylin and eosin (HE) staining showing fibrous thickening of the alveolar wall, glandular squamous metaplasia, and a lymphoid follicle structure caused by repeated pneumonitis

Operating time was 286 min, and blood loss was 100 ml. The chest tube was removed on postoperative day 3, and the patient was discharged soon thereafter. Macroscopic examinations of surgical specimens showed multiple cystic lesions with a boundary and elastic artery flow into that area (Fig. 3). Pathological examination findings revealed fibrous thickening of the alveolar wall, granular squamous metaplasia, and a lymphoid follicles structure as a result of repeated pneumonitis. Chest CT findings at 2 years after surgery showed good expansion of the residual left lower lobe with no consolidation and no thrombus in the stump of the vessel. Respiratory function was nearly the same as the preoperative condition.

### Discussion

Pulmonary sequestration was initially reported by Pryce in 1946 [1] and defined as a congenital abnormality of lung genesis and classified into three types based on the circulation area of the abnormal artery. ILS is performed to cover with the same pleura from the normal lung, while the extralobular type is treated using an independent pleura sample. ILS is commonly performed in the left S10, and the abnormal artery is branched from the thoracic aorta, followed by the abdominal aorta, whereas most of the circulating veins are pulmonary veins. The present case was a Price type 3 ILS, in which an abnormal artery flows only into sequestration tissue.

Chest CT findings are useful for diagnosis, as those can show cystic or consolidation areas with the abnormal artery mostly in the lower lobe. Importantly, three-dimensional CT findings can clearly reveal the vessel. Surgical resection is the first choice of treatment, because sequestration causes repeated inflammation even in asymptomatic patients. Generally, lobectomy is likely the most common procedure for ILS, because it is considered that complete resection of sequestration should be performed, and the border of normal part become unclear because surrounding tissues may be damaged due to repeated inflammation. Several reports of a lobectomy for thoracoscopic surgery have been presented [2–4], of which Liu noted no significant differences for operating time, blood loss, and morbidity as compared with a thoracotomy [2]. However, we consider that a sublobar resection is adequate if the lesion is included in the segment and it can be removed completely because of benign disease status. There are several reports of a segmentectomy, while there are a few known reported cases limited to thoracoscopic surgery [3, 5–7]. The present is the rare case of resection of one segment for an adult case and be able to show that good long-term prognosis.

Several methods are available to detect the border between segments, such as selective inflation [5] or injection of methylene blue from an abnormal vessel to reveal sequestration [6]. In our case, the border of sequestration was not clear, probably because of repeated inflammation, though we were able to confirm the sequestration included in S10 completely by selective inflation from B10. Chest CT performed after the operation showed good expansion of the residual lower lobe and no persistence of sequestration. Inflation of the specific segment of the adjacent sequestration was easily performed and useful to detect the cutting line.

However, many textbooks show that standard operation for ILS is lobectomy; we think that a segmentectomy of minimum segment performed under a thoracoscopic approach is effective and useful for intrapulmonary sequestration and should be considered as one of the options for this disease.

## Conclusions

A thoracoscopic segmentectomy for ILS is a feasible and useful procedure in qualified cases, even for adult patients.

## Abbreviations

B: Bronchus
CT: Computed tomography
HE: Hematoxylin and eosin
ILS: Intralobar sequestration
S: Segment
